# Characterization of dysregulated glutamine metabolism in human glioma tissue with ^1^H NMR

**DOI:** 10.1038/s41598-020-76982-7

**Published:** 2020-11-24

**Authors:** Selin Ekici, Benjamin B. Risk, Stewart G. Neill, Hui-Kuo Shu, Candace C. Fleischer

**Affiliations:** 1 Department of Radiology and Imaging Sciences, Emory University School of Medicine, Atlanta, GA 30322 USA; 2 Department of Biostatistics and Bioinformatics, Emory University, Atlanta, GA 30322 USA; 3 Department of Pathology and Laboratory Medicine, Emory University School of Medicine, Atlanta, GA 30322 USA; 4 Department of Radiation Oncology, Emory University School of Medicine, Atlanta, GA 30322 USA; 5 Wallace H. Coulter Department of Biomedical Engineering, Georgia Institute of Technology and Emory University, Atlanta, GA 30322 USA

**Keywords:** Tumour biomarkers, Biomarkers, Chemistry

## Abstract

Gliomas are one of the most common types of brain tumors. Given low survival and high treatment resistance rates, particularly for high grade gliomas, there is a need for specific biomarkers that can be used to stratify patients for therapy and monitor treatment response. Recent work has demonstrated that metabolic reprogramming, often mediated by inflammation, can lead to an upregulation of glutamine as an energy source for cancer cells. As a result, glutamine pathways are an emerging pharmacologic target. The goal of this pilot study was to characterize changes in glutamine metabolism and inflammation in human glioma samples and explore the use of glutamine as a potential biomarker. ^1^H high-resolution magic angle spinning nuclear magnetic resonance spectra were acquired from ex vivo glioma tissue (n = 16, grades II–IV) to quantify metabolite concentrations. Tumor inflammatory markers were quantified using electrochemiluminescence assays. Glutamate, glutathione, lactate, and alanine, as well as interleukin (IL)-1β and IL-8, increased significantly in samples from grade IV gliomas compared to grades II and III (*p* ≤ .05). Following dimension reduction of the inflammatory markers using probabilistic principal component analysis, we observed that glutamine, alanine, glutathione, and lactate were positively associated with the first inflammatory marker principal component. Our findings support the hypothesis that glutamine may be a key marker for glioma progression and indicate that inflammation is associated with changes in glutamine metabolism. These results motivate further in vivo investigation of glutamine as a biomarker for tumor progression and treatment response.

## Introduction

Gliomas are one of the most common classes of brain tumors, comprising 80% of malignant primary brain tumors^1,2^. Higher grade gliomas have a poor prognosis and high treatment resistance, due in part to a lack of specific and noninvasive treatment biomarkers^3,4^. While current diagnosis and treatment planning often includes radiological imaging [magnetic resonance imaging (MRI), computed tomography (CT), or positron emission tomography (PET)], grading and prognosis still rely on pathological results from invasive biopsies^2,5^. With the exception of 2-hydroxyglutarate (2-HG), there is an absence of specific and non-invasive imaging biomarkers for tumor grading, stratifying patients for targeted therapy, and monitoring treatment response^6,7^. The heterogeneous nature of gliomas, combined with increasing therapeutic strategies that target metabolic and immunologic pathways, motivates the need for non-invasive imaging strategies that provide molecular level information.

Cancer is increasingly recognized as a disease driven by metabolic reprogramming. The most well-known example is the Warburg effect, where neoplastic cells rely on aerobic glycolysis over more energy-efficient mitochondrial oxidative phosphorylation^8–10^. The Warburg effect induces a high glycolytic flux that maintains the adenosine triphosphate (ATP) reservoir needed for cancer cells to survive, but more importantly provides precursors for the biochemical building blocks such as nucleotides, amino acids, and lipids needed for proliferation^9–13^. In glioblastoma cells, it has been observed that up to 90% of cellular glucose is converted to lactate and alanine^11^. In comparison, studies in healthy murine brain tissue showed that ~ 7% of glucose is converted to lactate^11,14^. While glucose is a steady carbon source for fatty acid and energy substrates, it cannot provide the nitrogen that is needed for protein and nucleic acid synthesis, both of which are required for cancer proliferation.

Evidence suggests that this nitrogen demand is met with glutamine. Glutamine catabolism, or glutaminolysis, generates nitrogenous precursors for fatty acid, amino acid, and nucleotide synthesis^11,15,16^. Cancer cells can develop a dependence on glutamine for the nitrogenous biomass that is required for proliferation^17^. Increased glutamine concentration in tumor cells is associated with tumor growth, and as a result, pharmacologic strategies are increasingly targeting enzymes involved in glutaminolysis^15^. Promising targets include inhibiting glutaminase (GLS) and glutamate dehydrogenase (GDH), both of which catalyze the production of glutamate for use as an intermediate in the tricarboxylic acid (TCA) cycle^18–20^. Transaminase inhibitors are effective in preventing the conversion of glutamine to amino acids such as alanine that are then used in protein synthesis. As glutamine is a precursor for antioxidants such as glutathione, simultaneous inhibition of transaminases and glutathione production are promising potential treatments^21–23^.

Glutamine metabolism is also influenced by inflammation present in the tumor microenvironment due to oncogene activation and constitutive inflammatory cytokine production by tumor cells^10,24^. Inflammatory cells further aggravate metabolic dysregulation in cancer cells by activating transcription factors that upregulate glutamine transporters and the expression of enzymes including GLS and GDH^25–29^. As inflammation is an early indicator of tumor progression, the interplay between inflammation and metabolism may contribute to the high glutamine concentrations observed in proliferative gliomas^28,30^. Given the central role of glutamine in providing energy for cancer cells, and the recent increase in treatment strategies that target glutamine pathways, we hypothesize that glutamine may be a potential biomarker for prognosis, stratification, and treatment monitoring.

Clinically, in vivo quantification of metabolites such as glutamine can be performed using PET and MR spectroscopy (MRS). While PET facilitates direct imaging of metabolic processes, MRS provides a snapshot of multiple metabolites simultaneously without the use of ionizing radiation. Combined with the cost reduction, MRS is a more viable method for non-invasive and repeatable monitoring of disease progression and treatment. MRS studies have primarily focused on quantifying 2-HG, decreased levels of *N*-acetylaspartate (NAA), and increased choline in high grade gliomas^31,32^. However, despite the central role of glutamine in cancer metabolism and its potential as a new pharmacologic target, limited studies have explored the use of glutamine as a specific imaging biomarker. As a first step in identifying glutamine-associated metabolites as potential candidates for biomarker development, the aims of this study were to (1) quantify tumor metabolites in human glioma tissue using proton high-resolution magic angle spinning nuclear magnetic resonance (^1^H HRMAS NMR) spectroscopy, the ex vivo analog of MRS^33^; (2) quantify inflammatory markers in the tumor microenvironment using immunoassays; and (3) determine the relationships between glutamine and inflammation in human glioma tissue. ^1^H HRMAS NMR revealed increased concentrations of metabolites associated with glutaminolysis including glutamine, glutamate, glutathione, lactate, and alanine, and these metabolites were positively associated with pro-inflammatory cytokines in the tumor microenvironment. This multi-modal approach lays the groundwork for more comprehensive profiling of glutamine metabolism in gliomas, motivating future studies using in vivo MRS to non-invasively quantify glutamine in patients.

## Methods

### Human glioma tissue samples

This study used fully de-identified samples from human glioma patients and was deemed exempt by the Emory Institutional Review Board. Sixteen histologically-confirmed glioma samples [World Health Organization (WHO) grade II = 5; grade III = 6; grade IV = 5], collected from human brain tumor patients during surgical resection or excision and prior to radiation therapy or chemotherapy, were obtained from the Cancer Tissue and Pathology Biobank (Emory University Winship Cancer Institute). Inclusion criteria for the samples were patients ≥ 18 years old, containing > 40% tumor, and a diagnosis of diffuse astrocytoma, infiltrating astrocytoma, oligodendroglioma, anaplastic oligodendroglioma, anaplastic astrocytoma, glioblastoma, high grade astrocytoma, or high grade glioma. The final cohort included samples from eight males and eight females with an age range at the time of diagnosis of 28.4–71.5 years old (mean age ± standard deviation (SD) = 45.7 ± 12.9 years). Ten samples were from patients who were IDH-1 positive and one who was IDH-2 positive^34–36^. Tissue samples were flash frozen immediately after surgery and stored at − 80 °C prior to batch analysis.

### Electrochemiluminescence assays

Immunoassays were performed on tissue samples (≥ 10 mg) that were homogenized in 1× homogenization buffer [125 mM tris(hydroxymethyl)aminomethane, 15 mM MgCl_2_, 2.5 mM ethylenediaminetetraacetic acid, 1% Triton X-100, and protease inhibitor (Roche, 11697498001)]. Total protein was quantified with a bicinchoninic acid (BCA) assay (Pierce, 23225). Inflammatory markers were quantified in duplicate from tissue lysate using electrochemiluminescence assays performed according to the manufacturer’s instructions (Meso Scale Diagnostics, U-Plex Biomarker Assay, K15067L-1; V-Plex Human CRP Kit, K151STD-1). The mean concentration from duplicate assays was used in analysis and the following inflammatory markers were quantified: interleukin (IL)-1α, IL-1β, IL-8, IL-6, IL-10, IL-17A, interferon (IFN)-γ, tumor necrosis factor (TNF)-α, and C-reactive protein (CRP). Lower limits of detection for each inflammatory marker are shown in Supplementary Table S1.

### ^1^H HRMAS NMR spectroscopy

To prepare samples for solid state ^1^H HRMAS NMR, frozen tissue (10–15 mg) was aliquoted using a 2 mm biopsy punch (Braintree Scientific, Inc, MTP-33-31). Tissue was placed in an 80 μL HRMAS disposable insert (Bruker, B4493) inside a 4 mm zirconium oxide HRMAS rotor (Bruker, H14355). As tissue samples were retained and stored in the HRMAS NMR inserts for long-term stability analysis, separate aliquots of tissue were used for NMR spectroscopy and immunoassays. All NMR experiments were performed at 4 °C using a 600 MHz NMR spectrometer with an HRMAS probe (Bruker, AVANCE III). NMR spectra were acquired using the Carr–Purcell–Meiboom–Gill (CPMG, *cpmgr1d*) pulse sequence with a pre-saturation water suppression pulse and the following parameters: MAS spinning speed = 4000 Hz; complex data points = 16,384; spectral bandwidth = 8013 Hz; N = 512; and flip angle = 90°. The CPMG sequence consists of a 90° RF pulse that is followed by a train of spin echoes (delay-180°-delay, delay time = 1–2 ms). The 90° pulse was calibrated using a sample of sucrose in D_2_O prior to the HRMAS NMR experiments. Brain metabolite concentration ratios and Cramer–Rao lower bounds (CRLBs) were estimated using LCModel, a user-independent method for spectral quantification^37,38^. Spectra were analyzed between 3.85 and 0.2 ppm with a gamma-simulated, 26-metabolite basis set containing MR-detectable metabolites present in gliomas (alanine, ascorbate, aspartate, creatine, phosphocreatine, ethanolamine, γ-aminobutyric acid, glucose, glutamine, glutamate, glycine, glycerophosphocholine, phosphocholine, glutathione, 2-HG, myo-inositol, lactate, NAA, *N*-acetylaspartylglutamic acid, phosphoethanolamine, propylene glycol, *scyllo*-inositol, serine, taurine, valine, and acetate). Metabolite concentrations used for analysis were normalized to total creatine (creatine + phosphocreatine), and total creatine concentrations were normalized to water.

### Statistical analysis

Statistical analysis was performed with SPSS (IBM, v26.0) and R (v3.6.3). Metabolite ratios with CRLBs ≤ 30 were used for analysis. NMR spectra from two samples were unusable and were not included in the statistical analysis. Differences in metabolite and inflammatory marker concentrations as a function of grade (II, III, and IV) were determined using non-parametric Kruskal–Wallis H-tests followed by post-hoc pair-wise Dunn’s tests. A Bonferroni correction was applied to the *p* value of the Dunn’s tests to correct for multiple comparisons within a given Kruskal–Wallis test. Effect sizes (η^2^) were calculated from the H-statistic. For samples from patients with a known survival status, Mann–Whitney U-tests were performed to explore differences in metabolite and inflammatory marker concentrations as a function of survival.

To limit multiple comparisons of the detected inflammatory markers, probabilistic principal component analysis (PPCA) was used to reduce the dimensions of inflammatory marker concentrations and identify principal components (PCs)^39,40^. Concentrations below the detection limit of the immunoassays were imputed during PPCA, and inflammatory markers with fewer than ten observations were excluded. This resulted in the inclusion of IL-α, IL-1β, IL-8, and CRP, while TNF-α and IL-6 were excluded. Normality was assessed visually from histograms of inflammatory marker concentrations. As inflammatory marker concentrations were not normally distributed, data were log-transformed prior to PPCA. Two PCs were retained (cumulative R^2^ = .89). Each PC was comprised of loadings (a value from − 1 to 1) from individual inflammatory markers. For each NMR-quantified tumor metabolite that was significant in the omnibus Kruskal–Wallis H-test, a univariate regression was performed with metabolite as the response variable and the inflammatory marker PC as the predictor variable (PC-1 or PC-2). Significance for all analyses was determined by *p* ≤ .05.

## Results

Patient characteristics including grade and histopathological diagnosis for all tumor samples are shown in Table 1. A representative ^1^H HRMAS NMR spectrum and the corresponding LCModel fit are shown in Supplementary Fig S1^34,35^. Nine metabolites of interest were reliably detected and fit using LCModel (Supplementary Table S2).

**Table 1:** Characteristics of the glioma tissue samples.

Sample	Sex	Race	Age (years)^a^	Histologically confirmed diagnosis	WHO grade	Vital status	Treatment^b^	IDH mutation	% Tumor^c^
1	F	C	54	Oligodendroglioma	II	Alive	Unknown	IDH-1	60
2	F	C	45	Oligodendroglioma	II	Alive	RT + Chemo	IDH-1	80
3	F	C	35	Oligodendroglioma	II	Unknown	None	IDH-1	80
4	M	AA	45	Oligodendroglioma	II	Alive	RT + Chemo	IDH-1	90
5	M	AA	33	Diffuse astrocytoma	II	Unknown	RT; Chemo Unknown	IDH-1	85
6	M	C	36	Anaplastic oligodendroglioma	III	Alive	RT + Chemo	IDH-1	100
7	F	AA	28	Anaplastic Oligodendroglioma	III	Alive	RT + Chemo	IDH-2	75
8	F	C	38	Anaplastic astrocytoma with gemistocytic features	III	Alive	RT + Chemo	IDH-1	40
9	M	C	38	Anaplastic astrocytoma	III	Alive	RT + Chemo	IDH-1	95
10	F	C	36	Anaplastic oligodendroglioma	III	Alive	None	IDH-1	55
11	F	C	50	Anaplastic astrocytoma	III	Deceased	RT + Chemo	WT	95
12	F	C	50	Glioblastoma	IV	Deceased	None	WT	55
13	M	C	62	Glioblastoma	IV	Deceased	None	WT	80
14	M	C	70	Glioblastoma	IV	Alive	RT + Chemo	WT	70
15	M	AA	41	Residual glioblastoma with therapy-related changes	IV	Deceased	RT + Chemo	IDH-1	90
16	M	C	71	Glioblastoma	IV	Deceased	RT	WT	50

*M* male, *F* female, *C* caucasian, *AA* African American, *WHO* World Health Organization, *RT* radiation therapy, *Chemo* chemotherapy, *IDH* isocitrate dehydrogenase, *WT* wild type.

^a^Age at the time of diagnosis and sample collection.

^b^All patients underwent excision or resection prior to adjuvant treatment.

^c^Percentage of tissue sample that contained tumor, determined histologically.

**Figure 1:**
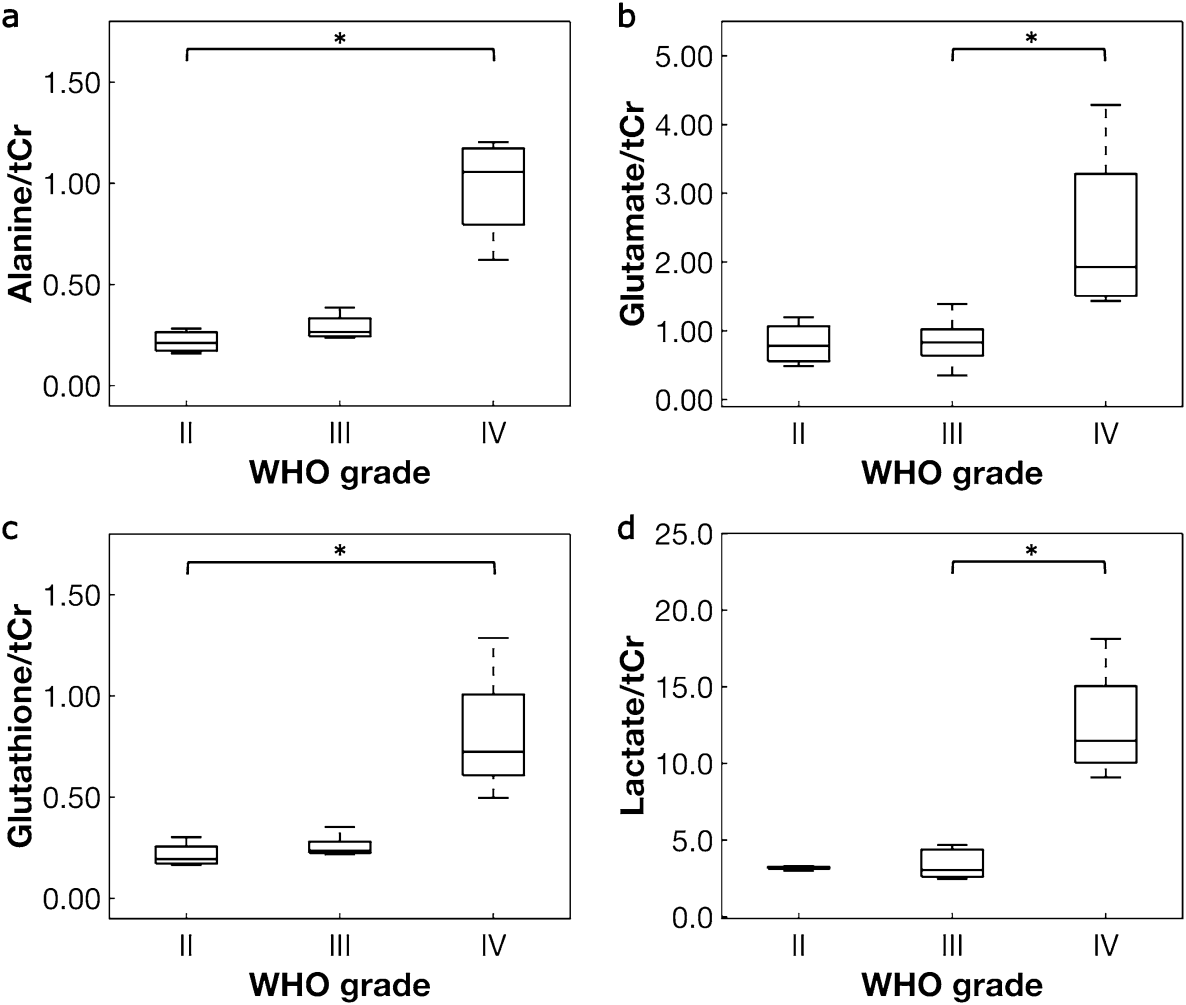
Box plots of tumor metabolites that varied significantly as a function of WHO grade. Metabolites in glioma samples were quantified using ^1^H HRMAS NMR. Differences as a function of WHO grade were assessed with Kruskal–Wallis H-tests and post-hoc pair-wise Dunn’s tests (Table 2). A Bonferroni correction was applied to the *p* value of the Dunn’s test to correct for multiple comparisons within each metabolite. Metabolites that varied significantly in post-hoc analysis include (**a**) alanine, (**b**) glutamate, (**c**) glutathione, and (**d**) lactate. Metabolite concentrations were normalized to creatine + phosphocreatine (tCr). *Denotes significance at *p* ≤ .05.

**Figure 2:**
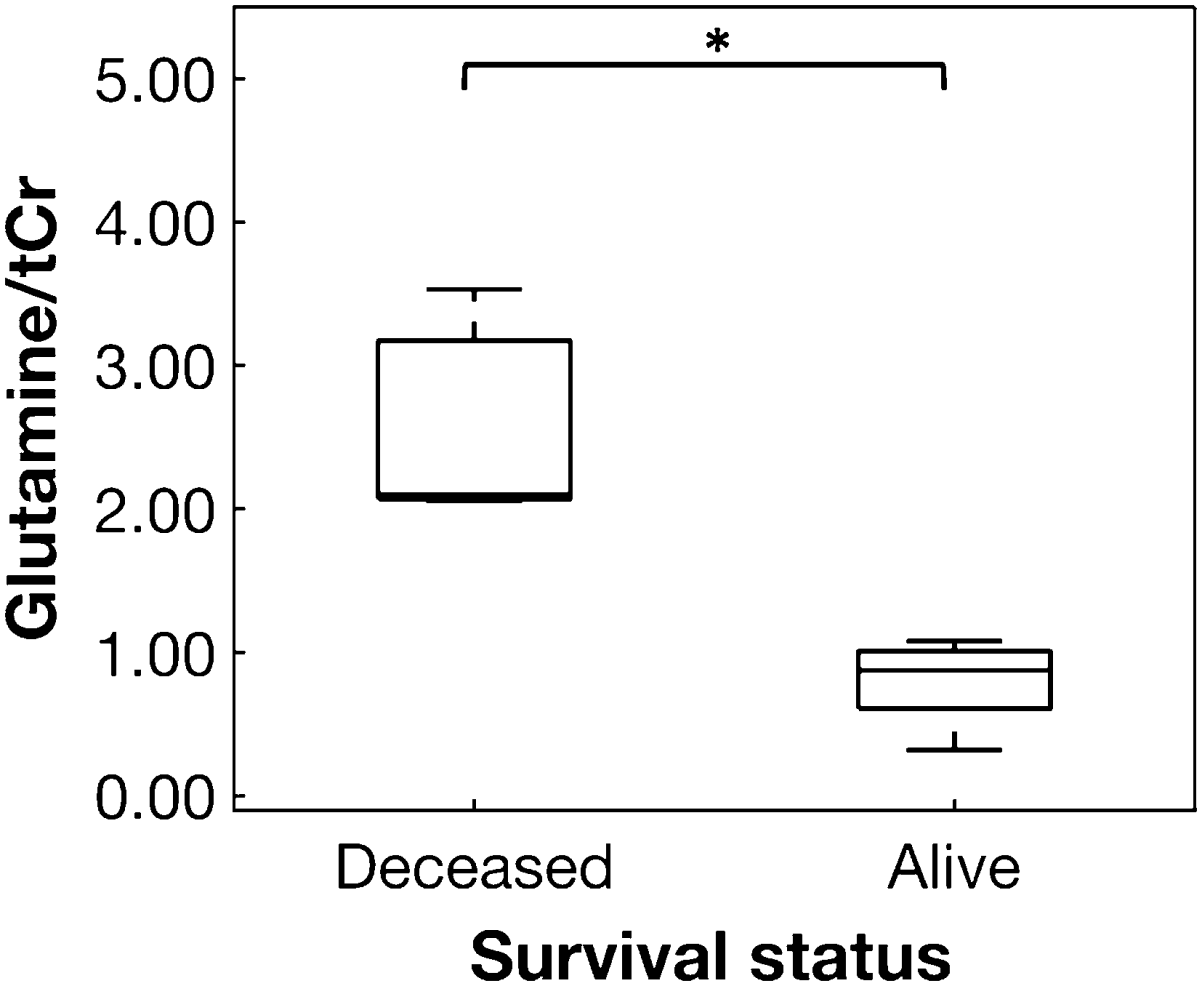
Glutamine concentration varied significantly as a function of survival status. Glutamine concentration was significantly higher in deceased versus alive patients when assessed with a Mann–Whitney U-test (*p* = .012). Glutamine was normalized to creatine + phosphocreatine (tCr). *Denotes significance at *p* ≤ .05.

Multiple tumor metabolites varied significantly as a function of WHO grade including alanine, glutamine, glutamate, glutathione, and lactate (Table 2)^36^. Effect sizes (η^2^) for the metabolite differences were large, ranging from .50 to .69. The remaining metabolites did not differ significantly between grades (Supplementary Table S3). Aspartate was only quantified in one sample and γ-aminobutyric acid in two samples. Importantly, total creatine (creatine plus phosphocreatine), normalized to water, was used to normalize the remaining metabolite concentrations and did not significantly vary between groups (*p* = .23). Post-hoc tests revealed that alanine and glutathione increased significantly in grade IV compared to grade II, and glutamate and lactate increased significantly in grade IV compared to grade III (Table 2, Fig. 1). Exploratory analysis revealed that alanine, glutamine, glutamate, glutathione, and lactate concentrations were significantly higher in samples from deceased patients compared to patients who are alive (*p* ≤ .05, Fig. 2). Mean survival time from diagnosis (when tissue samples were collected) to death was mean ± SD = 2.0 ± 2.5 years. For patients who are alive, mean survival time to date or at last contact was mean ± SD = 5.6 ± 4.7 years. Two patients had unknown survival status and were excluded from the exploratory analysis. Most samples from deceased patients were grade IV compared to variable tumor grades observed in patients who are alive (Table 1). Of note, while most patients received adjuvant radiation therapy plus chemotherapy, two patients who are deceased and one who is alive did not receive treatment after surgical resection.

**Table 2:** Differences in tumor metabolite concentrations as a function of WHO grade.

Metabolite^a^	H^b^	*p* value^*c*^	η^2,d^	Post-hoc comparisons (*p* values)^e^		
				II versus III	III versus IV	II versus IV
Alanine/tCr	7.477	**.024**	.69	> .99	.13	**.031**
Glutamine/tCr	6.522	**.038**	.50	> .99	.073	.10
Glutamate/tCr	7.714	**.021**	.57	> .99	**.039**	.055
Glutathione/tCr	8.782	**.012**	.68	.90	.13	**.011**
Lactate/tCr	8.051	**.018**	.55	> .99	**.028**	.051

tCr = creatine + phosphocreatine.

^a^Metabolite concentrations were normalized to tCr.

^b^Non-parametric Kruskal–Wallis H-test.

^c^Bolded values indicate statistical significance (*p* ≤ .05).

^d^Effect size was calculated using η^2^.

^e^*p* values adjusted using the Bonferroni correction for comparing tumor grade within metabolite.

**Figure 3:**
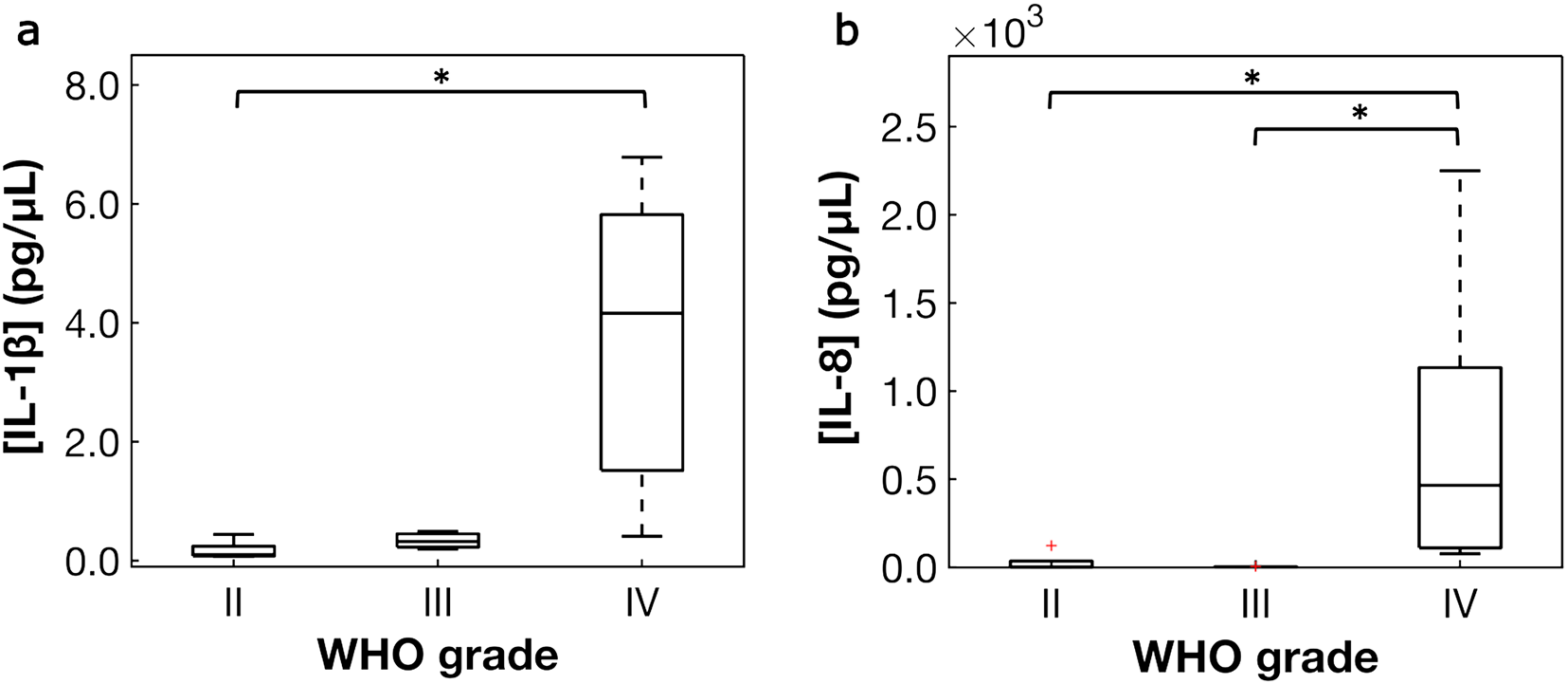
Box plots of inflammatory markers that varied significantly as a function of WHO grade. Inflammatory markers in glioma samples were quantified using electrochemiluminescence assays. Differences as a function of WHO grade were assessed with Kruskal–Wallis H-tests and post-hoc pair-wise Dunn’s tests (Table 3). A Bonferroni correction was applied to the *p* value of the Dunn’s test to correct for multiple comparisons within each inflammatory marker. Significant differences were observed for (**a**) IL-1β, and (**b**) IL-8. *Denotes significance at *p* ≤ .05.

**Figure 4:**
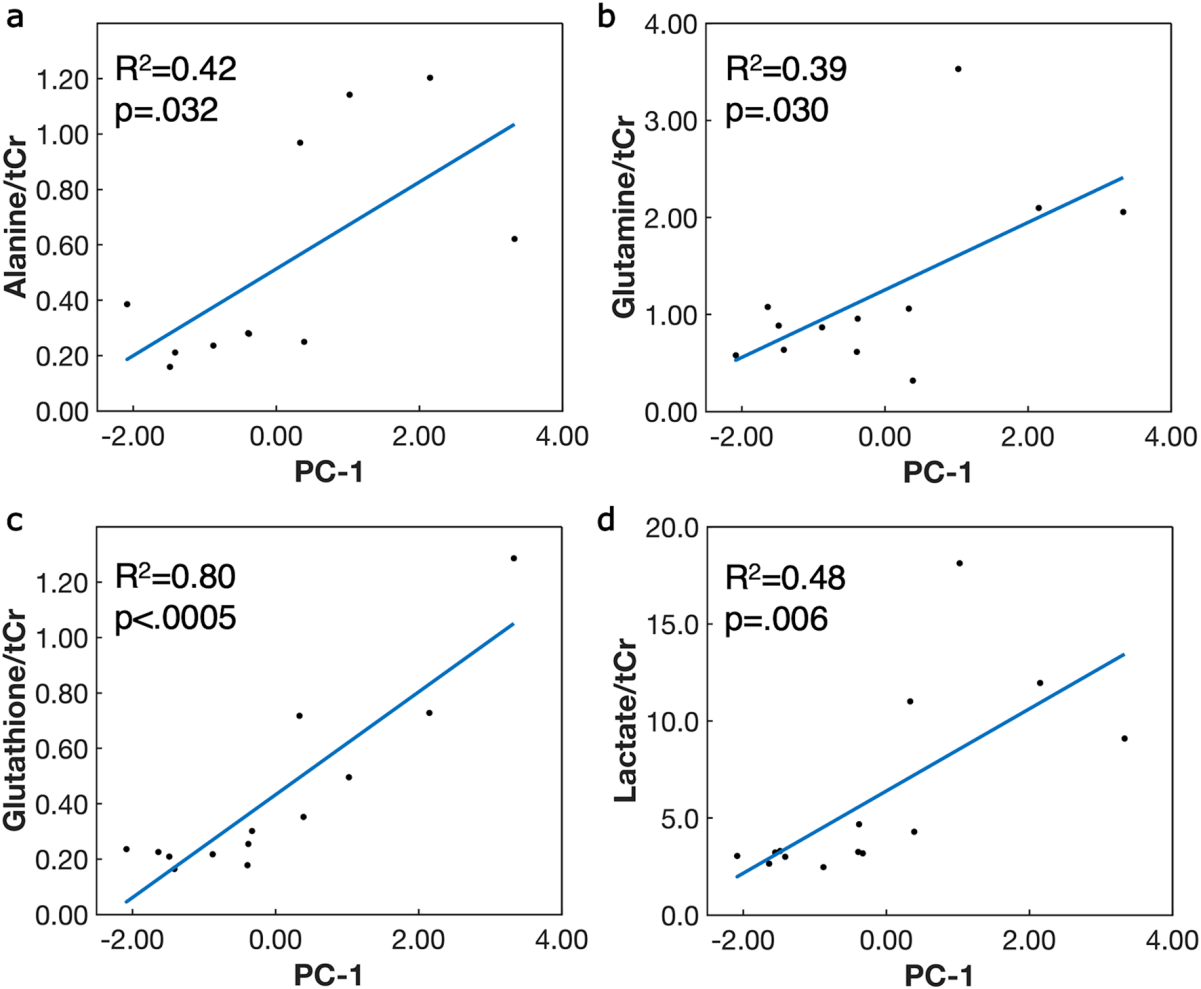
Significant associations between tumor metabolite concentrations and inflammatory marker principal component-1 (PC-1). PC-1 contains contributions primarily from interleukin (IL)-1α, IL-1β, and IL-8. Solid black circles represent raw data and blue line is the linear least-squares regression. Significant positive associations were observed between (**a**) alanine, (**b**) glutamine, (**c**) glutathione, and (**d**) lactate with inflammatory marker PC-1. Metabolites were normalized to creatine + phosphocreatine (tCr). Significance was determined by *p* ≤ .05.

Inflammatory markers including IL-1α, IL-1β, IL-6, and IL-8 varied significantly with WHO grade (Table 3), while CRP did not (*p* > .05, Supplementary Table  S4)^36^. Concentrations of IL-10, IL-17A, and IFN-γ were below the detection limits for all samples. From the post-hoc analysis, IL-8 increased significantly from grade II to grade IV and from grade III to grade IV, and IL-1β increased from grade II to IV (Fig. 3). IL-1α and IL-6 did not vary significantly in the post-hoc analysis (Table 3). Exploratory analysis as a function of survival status revealed higher concentrations of IL-1α, IL-1β, and IL-8 in samples from deceased patients compared to those who are alive. PPCA resulted in two significant inflammatory marker PCs. PC-1 contained contributions from three inflammatory markers with loadings > .5 (IL-1α, IL-8, IL-1β), and contributions in PC-2 were primarily from CRP (loading = − .92, Supplementary Table  S5)^36^. Univariate PC regressions revealed significant, positive relationships between alanine, glutamine, glutathione, and lactate with PC-1, while no metabolites were significantly associated with PC-2 (Fig. 4, Supplementary Table S6)^36^. The association of glutamine with PC-1 was nearly significant (*p* = .051). The remaining metabolites were not significantly associated with either of the inflammatory marker PCs (*p* > .05).

**Table 3:** Differences in tumor inflammatory marker concentrations as a function of WHO grade.

Inflammatory marker	H^a^	*p* value^b^	η^2^^c^	Post-hoc comparisons (*p* values)^d^		
				II versus III	III versus IV	II versus IV
IL-1α	6.256	**.044**	.35	> .99	.11	.062
IL-1β	8.105	**.017**	.61	.84	.50	**.013**
IL-6	6.400	**.041**	.73	> .99	.14	.14
IL-8	8.346	**.015**	.49	> .99	**.030**	**.042**

*IL* interleukin.

^a^Non-parametric Kruskal–Wallis H-test.

^b^Bolded values indicate statistical significance (*p* ≤ .05).

^c^Effect size was calculated using η^2^.

^d^*p* values adjusted using the Bonferroni correction for comparing tumor grade within inflammatory marker.

## Discussion

Glutamine is increasingly recognized as a vital biomolecule for cancer metabolism, and emerging pharmacologic strategies are being developed that target glutamine-associated metabolic pathways^41^. In this pilot study, ^1^H HRMAS NMR and immunoassays were used to investigate the relationship between glutamine metabolism and inflammation as a first step in exploring glutamine as an NMR-detectable biomarker of glioma progression. Previous ^13^C NMR spectroscopy studies have observed that proliferating glioblastoma cells in murine brains accumulate large concentrations of ^13^C-labelled glutamine as a result of increases in both glutamine uptake and synthesis from oxidized glucose^11,42^. These labeled carbon atoms from glutamine are then incorporated into glutamate and alanine during early stages of glutaminolysis. Consistent with these findings, we observed significant changes in glutamine-associated metabolites as a function of WHO grade. Glutamine, glutamate, alanine, lactate, and glutathione all varied significantly as a function of tumor grade. Post-hoc pair-wise analysis revealed higher concentrations in samples from grade IV gliomas compared to grades II and III, supporting the hypothesis that glutamine metabolism, particularly glutaminolysis, is dysregulated in gliomas. Interestingly, while several studies have reported changes in choline and NAA as a function of tumor grade^31,43,44^, we did not observe changes in these metabolites. Saindane et al. examined the efficacy of MRS to differentiate metabolites in high grade gliomas from non-cancerous brain lesions and similarly reported non-significant differences in choline, NAA, and lactate concentrations^45^. As choline and NAA are unlikely to differentiate glioma metabolism from other brain pathologies, glutamine-associated metabolites may be more viable biomarker candidates for differentiating tumors and informing treatment.

Importantly, glutamine-associated metabolites, as well as several inflammatory markers, were also significantly higher in glioma samples from patients who are since deceased compared to patients who are alive. While exploratory, this is consistent with our hypothesis that increased glutamine concentration may be an indicator of poor prognosis. As several patients (two deceased and one who is alive) did not receive adjuvant treatment after surgical resection, treatment effects likely contribute to these observations. It will be important for future studies to identify the relationship between changes in glutamine metabolism and response to treatment and determine if glutamine metabolites are potential biomarkers for patient stratification and prognosis.

Not surprisingly, we observed an increase in inflammatory marker concentrations with WHO grade, particularly IL-1α, IL-1β, IL-6, and IL-8. Pro-inflammatory cytokines IL-1β, IL-6 and IL-8 are expressed in the tumor microenvironment and promote tumor growth^46^. In astrocytoma cells, IL-1α contributes to the transcription and activation of IL-8 and IL-6, thereby inducing chronic inflammation^47^. IL-6 is also associated with decreased survival in resected glioma tissue, and may be a prognostic indicator for glioma patients^48^. Notably, all of our samples had > 40% tumor and most did not contain necrotic tissue, however, the presence of necrosis may have contributed to the higher inflammatory marker concentrations observed in grade IV samples. Further exploration into the mechanisms that drive changes in inflammatory cytokines within the tumor microenvironment are warranted.

While many reports have demonstrated that metabolites and inflammatory markers are altered in gliomas, our multimodal approach revealed significant associations between inflammation and NMR-detectable metabolites. Inflammation in the tumor microenvironment activates transcription factors including nuclear factor kappa-light-chain-enhancer of activated B cells (NF-κB) and hypoxia-inducible factor (HIF)-1^24^. Upon activation, NF-kB increases the expression of glucose transporters and GLS, enabling high rates of glycolysis and glutaminolysis^49^. Moreover, under conditions of hypoxia and high intracellular glutamine, inflammatory transcription factor HIF-1 increases the expression of glutamine transporters, glycolytic enzymes, and amino acid transporters^50–52^. Our results are consistent with these reports as we observed significant correlations between glutamine, alanine, glutathione, and lactate with inflammatory marker PC-1. PC-1 included contributions from IL-8, IL-1β, and IL-1α, inflammatory markers that upregulate NF-κB and HIF-1 in glioblastomas^53^. Interestingly, there were no significant relationships between CRP and any of the metabolites. CRP is produced by liver cells and acts as a systemic marker of inflammation in the blood. Cancer patients often exhibit high levels of CRP in plasma, however there is limited evidence that it accumulates in the tumor microenvironment^54^. Our results suggest that tumor levels of CRP may not be linked to metabolic dysregulation, or that the influence of CRP on metabolism may be different from other pro-inflammatory cytokines.

This study has several limitations. First, the sample size is small, which limited statistical power. In particular, we were not able to correct for multiple comparisons across metabolites due to potential collinearity, as this leads to unreasonable loss of power. However, we note that the effect sizes for metabolite changes as a function of grade are quite large (η^2^ ≥ .50 for all metabolites). Our exploratory analysis revealed differences as a function of survival status, but these results must be further verified with a larger population that considers treatment effects. The relationship between glutamine-associated metabolite changes and IDH status is also an important yet outstanding question for future studies. Our analysis used metabolites that were normalized to total creatine to facilitate comparisons. While we did not observe changes in total creatine as a function of tumor grade, this needs to be determined explicitly for each study or a non-internal reference can be used. Additionally, ^1^H HRMAS NMR may not have captured the metabolic variation across the assessed tumors, and separate aliquots of tissue were used for NMR and immunoassay analyses. Gliomas are highly heterogenous, with different regions of the same tumor displaying distinct molecular and genetic phenotypes^3^. Sampling bias effects should be considered in future studies, and whole tumor MRS may be able to discriminate metabolic heterogeneity among tumors and indicate appropriate treatments for patients with heterogenous tumors^55^. Finally, resection can induce metabolic changes in the tumor tissue, and future in vivo validation is particularly important for metabolites such as lactate that may be elevated during or after surgery.

In conclusion, we present a multi-modal approach to study the tumor microenvironment that utilizes ^1^H HRMAS NMR and inflammatory marker quantification. Glutamine-associated metabolites, as well as several pro-inflammatory cytokines, were significantly increased as a function of tumor grade and were positively associated with tumor inflammatory markers. Given the increase in treatments that target glutamine pathways, glutamine is a potential biomarker for stratifying patients for therapy and monitoring treatment response^15,21^. Glutamine can be quantified non-invasively in the healthy human brain using in vivo MRS. As we observed increased concentrations of glutamine-associated metabolites in glioma tissue, these results support further study in patients to determine if similar trends are observed in vivo. Future work will translate our ex vivo results to in vivo MRS studies with the goal of facilitating non-invasive diagnosis, prognosis, and treatment response monitoring in gliomas.

## Supplementary information

Supplementary information.

## Data Availability

Raw NMR spectra are available upon request after a data sharing agreement is executed between the respective institutions.
