# Supplementary material for: Characterization of dysregulated glutamine metabolism in human glioma tissue with 1H NMR
Source: Sci Rep. 2020 Nov 24;10:20435. doi: 10.1038/s41598-020-76982-7 (PMC7686482; doi:10.1038/s41598-020-76982-7)
Supplement: Supplementary file 1 — Supplementary information. [file 41598_2020_76982_MOESM1_ESM.docx]

**Characterization of dysregulated glutamine metabolism in human glioma tissue with ^1^H NMR**

Selin Ekici,^1^ Benjamin B. Risk,^2^ Stewart G. Neill,^3^ Hui-Kuo Shu,^4^ Candace C. Fleischer^1, 5*^

**Supplementary Material**

Supplementary Material includes Figure S1 and Tables S1-S6.


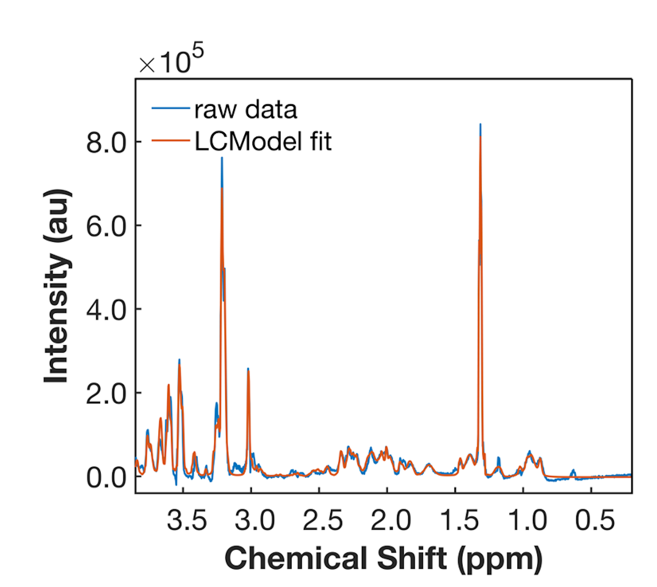


**Figure S1. Representative ^1^H HRMAS NMR spectrum and LCModel fit.** Raw data (blue) was acquired using a 600 MHz solid state HRMAS NMR spectrometer, analyzed from 3.85 – 0.2 ppm, and fit to a 26-metabolite basis set using LCModel (orange).

**Table S1. Lower limits of detection for inflammatory marker assays.**

| **Inflammatory Marker** | **Detection Limit (pg/μL)** |
| --- | --- |
| CRP | 1.33 |
| IFN-γ | 1.7 |
| IL-1α | 0.98 |
| IL-1β | 0.15 |
| IL-6 | 0.44 |
| IL-8 | 0.15 |
| IL-10 | 0.14 |
| IL-17A | 2.6 |
| TNF-α | 0.54 |
| CRP = C-reactive protein; IFN = interferon; IL = interleukin; TNF = tumor necrosis factor | |

Table S2. Mean CRLBs for metabolites quantified from ^1^H HRMAS NMR spectra.

| **Metabolite** | **No. spectra with detected metabolite** | **CRLB** |
| --- | --- | --- |
| Alanine | 11 | 17.5 |
| Glutamine | 12 | 10.0 |
| Glutamate | 13 | 10.8 |
| Glutathione | 12 | 16.4 |
| Lactate | 14 | 4.6 |
| tNAA | 9 | 12.8 |
| Myo-inositol | 14 | 8.0 |
| tCr | 14 | 8.7 |
| tCho | 13 | 8.8 |
| tNAA = *N*-acetylaspartate + *N*-acetylaspartylglutamic acid; tCr = creatine + phosphocreatine; tCho = glycerophosphocholine + phosphocholine; CRLB = Cramer Rao lower bound | | |

**Table S3. Differences in tumor metabolite concentrations as a function of WHO grade evaluated with the Kruskal Wallis H-test.**

| **Metabolite^a^** | **H-test**  **(*p-value*)^b^** |
| --- | --- |
| Alanine/tCr | **.024** |
| tCr/H_2_O | .23 |
| Glutamine/tCr | **.038** |
| Glutamate/tCr | **.021** |
| tCho/tCr | .22 |
| Glutathione/tCr | **.012** |
| 2-HG/tCr | .67 |
| Lactate/tCr | **.018** |
| Myo-inositol/tCr | .59 |
| tNAA/tCr | .13 |
| tCr = creatine + phosphocreatine; GABA = γ-aminobutyric acid; tCho = glycerophosphocholine + phosphocholine; 2-HG = 2-hydroxyglutarate; tNAA = *N*-acetylaspartate + *N*-acetylaspartylglutamic acid | |
| ^a^ Metabolite concentrations were normalized to tCr; tCr was normalized to water | |
| ^b^ Bolded values indicate statistical significance (p≤.05) | |

**Table S4. Differences in tumor inflammatory marker concentrations as a function of WHO grade evaluated with the Kruskal Wallis H-test.**

| **Inflammatory Marker** | **H-test**  **(*p-value*)^a^** | |
| --- | --- | --- |
| CRP | .63 | |
| IL-1α | **.044** | |
| IL-1β | **.017** | |
| IL-6 | **.041** | |
| IL-8 | **.015** | |
| CRP = C-reactive protein; IL = interleukin | |  |
| ^a^ Bolded values indicate statistical significance (p≤.05) | |  |

**Table S5. Inflammatory marker loadings onto individual principal components.**

| **Inflammatory Marker** | **PC-1** | **PC-2** |
| --- | --- | --- |
| IL-1β | 0.56 | 0.18 |
| IL-8 | 0.55 | 0.34 |
| IL-1α | 0.53 | 0.0042 |
| CRP | 0.31 | -0.92 |
| IL = interleukin; CRP = C-reactive protein; PC = principal component | | |

**Table S6. Univariate linear regression analysis of metabolite concentrations that varied significantly with grade as a function of inflammatory principal component scores.**

| **Metabolite^a^** | **PC-1** | | **PC-2** | |
| --- | --- | --- | --- | --- |
|  | **β^b^ ± SE** | ***p-value*^c^** | **β ± SE** | ***p-value*** |
| Alanine/tCr | 0.64±0.06 | **.032** | 0.39±0.12 | .24 |
| Glutamine/tCr | 0.62±0.14 | **.030** | 0.26±0.28 | .42 |
| Glutamate/tCr | 0.55±0.16 | .051 | 0.30±0.31 | .31 |
| Glutathione/tCr | 0.89±0.03 | **<.0005** | 0.07±0.10 | .82 |
| Lactate/tCr | 0.69±0.64 | **.006** | 0.30±1.35 | .29 |
| PC = principal component; SE = standard error; tCr = creatine + phosphocreatine | | | | |
| ^a^ Metabolite concentrations were normalized to tCr | | | | |
| ^b^ Standardized coefficients and standard error | | | | |
| ^c^ Bolded values indicate statistical significance (p≤.05) | | | | |
